# BMAL1—Potential Player of Aberrant Stress Response in Q31L Mice Model of Affective Disorders: Pilot Results

**DOI:** 10.3390/ijms252212468

**Published:** 2024-11-20

**Authors:** Kristina Smirnova, Tamara Amstislavskaya, Liudmila Smirnova

**Affiliations:** 1 Research Institute of Mental Health, Tomsk National Research Medical Center, Russian Academy of Sciences, Aleutskaja, 4, 634014 Tomsk, Russia; lpsmirnova2016@gmail.com; 2 Research Institute of Neuroscience and Medicine, Timakova 4, 630090 Novosibirsk, Russia; amstislavskayatg@neuronm.ru

**Keywords:** DISC1, BMAL1, stress, affective disorders, depression, Q31L mice

## Abstract

Dysregulation in the stress-response system as a result of genetical mutation can provoke the manifestation of affective disorders under stress conditions. Mutations in the human *DISC1* gene is one of the main risk factors of affective disorders. It was known that DISC1 regulates a large number of proteins including BMAL1, which is involved in the regulation of glucocorticoid synthesis in the adrenal glands and the sensitivity of glucocorticoid receptor target genes. Male mice with a point mutation Q31L in the *Disc1* gene were exposed to chronic unpredictable stress (CUS), after which the behavioral and physiological stress response assessed. To assess whether there were any changes in BMAL1 in key brain regions involved in the stress response, immunohistochemistry was applied. It was shown that the Q31L mice had an aberrant behavioral response, especially to the 2 weeks of CUS, which was expressed in unchanged motor activity, increased time of social interaction, and alterations in anxiety and fear-related behavior. Q31L males did not show an increase in blood corticosterone levels after CUS and a decrease in body weight. Immunohistochemical analysis in intact Q31L mice revealed a decrease in BMAL1 immunofluorescence in the CA1 hippocampal area and lateral habenula. Thus, the Q31L mutation of the *Disc1* gene disrupts behavioral and physiological stress response and the BMAL1 dysregulation may underlie it, so this protein can act as a molecular target for the treatment of affective disorders.

## 1. Introduction

Affective disorders such as depression or bipolar affective disorder are highly heritable psychiatric conditions and have strong genetic components, as evidenced by twin, adoption, and family studies [1]. Depression is a widespread mental disorder with high recurrence and low remission rates, leading to a significant decrease in life quality and a disruption of social functioning [2]. Mutations in the human *DISC1* gene are one of the main risk factors of mood disorders such as bipolar and major depressive disorders and schizophrenia [3]. The formation of abnormal DISC1 protein forms is accompanied by neuronal networks disruption, synaptic transmission, and neuronal integration anomalies that are involved in the regulation of cognitive and affective processes, and can cause the development of psychopathologies [3,4,5,6]. However, in the pathogenesis of mental disorders, not only are genetics involved, but also environmental factors which induce stress [7,8,9].

The *Disc1* gene and its protein have some conservativity in vertebrates including humans and rodents [10]. The *Disc1* exon sizes in mice are mostly similar to the human *DISC1* genomic structure. Exon 2 is the largest exon in *Disc1*, with 992 bp, and exon 2 is also the largest exon in human *DISC1*, with 980 bp. In addition, the smallest exon, exon 7, is also the same in mice and humans. Exons 3, 6, 7, 8, 9, 10, and 12 are the same size in mice and humans [11]. The similarity of the amino acid sequence of the DISC1 protein in humans and rodents is about 50–60% in the globular N-region and 75% in the helical C-region, and the first two exons of the *Disc1* gene always encode the N-terminal domain and a conserved nuclear localization signal. This has allowed for numerous models to be created with the *Disc1* gene mutation to study the mechanisms of affective disorder development [12]. These genetic models, without any environmental intervention, show some abnormalities that are relevant for affective disorders in human such as parvalbumin and synaptic deficits, enlarged lateral ventricles, disturbed dopaminergic system, and cognitive and emotional behavioral deficits [13].

Mice obtained with ENU-mutagenesis on a C57BL/6 background with point A/T transversion in 127 positions in the second exon led to the replacement of 31 glutamine (Q) amino acids in the DISC1 protein with leucine (L). The resulting mouse line was named Disc1-Q31L (Q31L). These mice were characterized by a depressive-like phenotype expressed in behavioral despair, low rates of social interaction, decreased sucrose preference, and deficit in working memory, all improved by antidepressants [14]. A later study described anxiety in males, increased emotionality and impulsivity in females, and longer extinction of fear memory compared to wild-type mice in mice of both sexes [15,16]. In the Q31L mice, impaired proliferation and the localization of neuronal precursors were found as well as decreased density and changes in the dendritic spines’ morphology in the frontal cortex and hippocampus, but an increased density of neurons was found in the lateral and medial habenula (LHb and MHb) [15,17]. Q31L mutation led to a decrease in dopamine, serotonin, norepinephrine, β-arrestin-1,2 and CREB levels in the nucleus accumbens, an increase in the level of pro-inflammatory cytokines in the frontal cortex and striatum, a decrease in the hippocampus and hypothalamus, and the content of anti-inflammatory interleukins was increased in the frontal cortex and decreased in the hippocampus [18,19]. These abnormalities affect brain areas such as the hippocampus and habenula, which are involved in the regulation of the stress response [20,21]. In this case, the Q31L mice may have had an inadequate reaction on the environmental stimuli, which complicate the adaptation and cause the development or manifestation of mood disorders under stress conditions [22]. It has previously been shown that heterozygous Q31L mice under a chronic social defeat stress paradigm demonstrated a weak stress response, but the mechanism of this gene–environment interaction is still not understood [23]. Thus, studying new stress-response mechanisms on a genetic model of depressive-like phenotype—Q31L mice could help find new therapeutic aims for curing stress-associated affective disorders. 

In Q31L mice, a disruption of the DISC1 N-terminus interactome including decrease interaction between DISC1 and glycogen sintaskinase-3 (GSK-3) has been shown, which is accompanied by increased GSK-3 phosphorylation activity [6]. Recently, Lee S.B. et al. (2021) showed that the 1–3 exon deletion of *Disc1,* which encodes the N-terminus of the DISC1 protein in embryonal cells, led to increased GSK-3-dependent BMAL1 (basic helix-loop-helix ARNT-like protein 1) degradation [24]. BMAL1 is a transcriptional factor that is widespread in many tissues and cells and determines the rhythmic activation of numerous genes including genes involved in stress response (*Fkbp5*, *Avp*, etc.) [25,26]. It has been shown that BMAL1 plays a role in glucocorticoid synthesis by increasing the adrenal cortex sensitivity to adrenocorticotropic hormone (ACTH) and regulating steroid synthesis [27,28]. Moreover BMAL1 can suppress the transcription of genes carrying glucocorticoid-responsive elements (GREs) on their promoters, which confirms the role of this protein in regulating cell sensitivity to glucocorticoids [28,29]. All of these indicate the role of this protein in the regulation of stress response.

**Figure A1:**
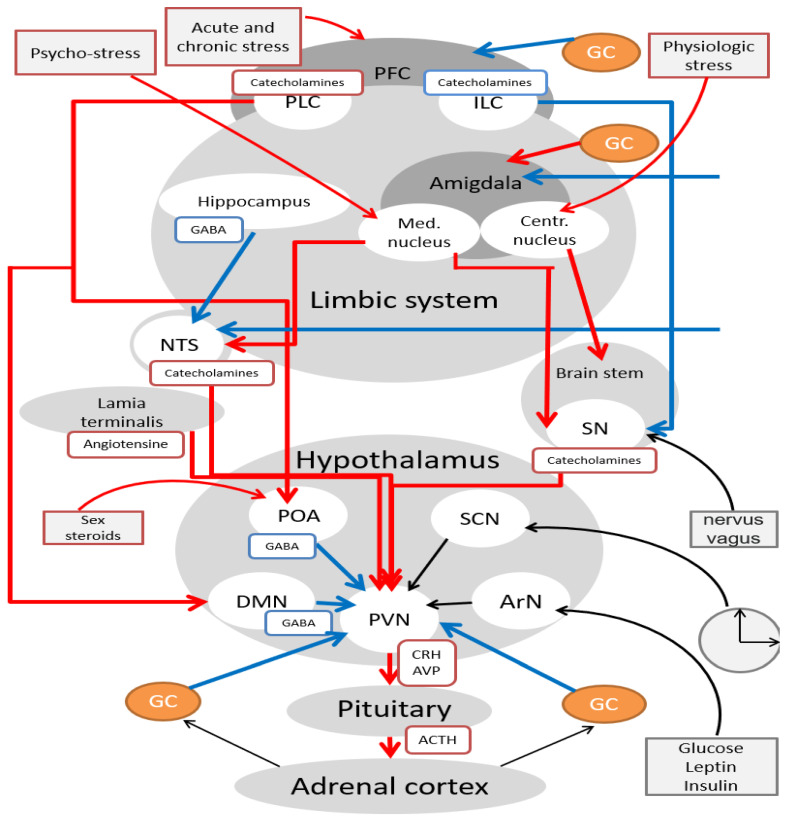
Neuroendocrine components of the stress-response system. Picture based on Smith, S.M. and Vale, W.W. (2006) [20] Black arrows—neutral or cyclical influences; blue arrows—inhibitory influences; red arrows—activating influences. Abbreviations: AVP—arginine vasopressin; ACTH—adrenocorticotropic hormone; GABA—gamma-aminobutyric acid; GC—glucocorticoids; ArN—arcuate nucleus; DMN—dorsomedial nucleus of the hypothalamus; ILC—infralimbic cortex; CRH—corticotropin releasing hormone; PVN—paraventricular nucleus of the hypothalamus; PLC—perilimbic cortex; POA—preoptic area; PFC—prefrontal cortex; SCN—suprachiasmatic nucleus; NTS—nuclei of the terminal strip; SN—Nucleus of the solitary tract; Psycho. stressors—psychological stressors.

In this study, we subjected homozygous Q31L mice to the chronic unpredictable stress (CUS) paradigm of varying duration (2 and 4 weeks) to define behavioral, hormonal, and physiological stress response in these mice. Due to the fact that the Q31L mutation also affects the N-terminus and increases GSK-3 phosphorylation activity, we additionally assessed the presence of BMAL1 in key brain structure such as the hippocampus, amygdalar complex, habenula, and suprachiasmatic nucleus (SCN) of intact Q31L mice, which are involved in affective disorders and the regulation of the hypothalamic-pituitary-adrenal (HPA axis, Figure A1), to assess molecular changes in the stress-response system caused by *Disc1* mutation.

## 2. Results

### 2.1. Two Weeks of CUS Decreased WT Mice Locomotor Activity but Had No Effect on Q31L Mice

**Figure 1:**
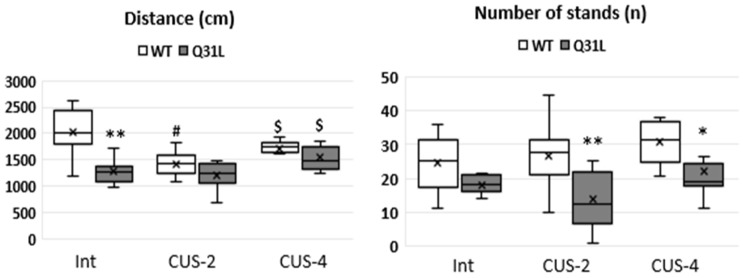
Locomotor activity parameters in the OF test. Data presented as the median, Q1 and Q3, minimum and maximum as whiskeys, x—mean; Int—intact animals, CUS-2—animals after two weeks of CUS, CUS-4—animals after four weeks of CUS; *^,#,$^ *p* < 0.05, ** *p* < 0.01. *—significant difference between genotypes in subgroups, #—significant difference between CUS subgroup and Int inside one genotype, $—significant difference between CUS-2 and -4 inside one genotype. Sample size: 8–10 mice in each group.

Locomotor activity was assessed by the open field (OF) test (Figure 1). There was a difference between the WT (wild-type or C57BL/6 mice) and Q31L mice in traveled distance only in the Int subgroup (M-U, *p* < 0.01). Analysis by the Kruskal–Wallis test of the distance traveled revealed differences between groups within the WT (χ^2^ = 11.17; df = 2; *p* < 0.01) and Q31L (χ^2^ = 7.44; df = 2; *p* < 0.05) genotypes. Comparison of the M-U criterion showed the significantly reduced locomotor activity of WT mice (M-U, *p* < 0.01) after two weeks of CUS (CUS-2). After 4 weeks of CUS (CUS-4) the WT mice traveled a significantly higher distance than the WT mice after CUS-2 (M-U, *p* < 0.05), but stayed in intact in the WT range. After 2 and 4 weeks of CUS, the WT animals reached the locomotor activity level of the Q31L mice in all subgroups. Stress did not affect the distance traveled in the Q31L mice compared with the Int subgroup, but even after the CUS-4 Q31L mice traveled a significantly higher distance than the Q31L mice after CUS-2 (M-U, *p* < 0.05), both subgroups stayed within the intact Q31L range. 

### 2.2. CUS Did Not Affect Anxiety but Changed Anxiety-Related Behavior and Induced Freezing in Q31L

**Figure 2:**
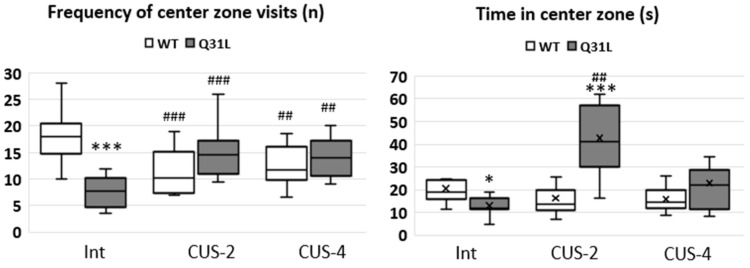
Anxiety related parameters of the OF test. Data presented as median, Q1 and Q3, minimum and maximum as whiskeys, x—mean; Int—intact animals, CUS-2—animals after two weeks of CUS, CUS-4—animals after four weeks of CUS; * *p* < 0.05, ^##^ *p* < 0.01, ***^, ###^ *p* < 0.001; *—significant difference between genotypes in subgroups. Sample size: 8–10 mice in each group.

Anxiety was assessed with the OF test and dark light chamber (DLC) test with unpredictable results. ANOVA analysis of the OF test center zone visiting frequency showed a significant interaction of genotype and stress factors (F [2,51] = 15.1, *p* < 0.001). Q31L mice in the Int subgroup visited the center zone rarely compared to the WT mice (LSD, *p* < 0.05); after CUSs, these differences were erased. While CUSs led to a decrease in the number of visits in the WT (LSD, *p* < 0.001 and *p* < 0.01 for CUS-2 and CUS-4, respectively), in Q31L, is was possible to see the opposite effect (LSD, *p* < 0.001 and *p* < 0.01 for CUS-2 and CUS-4, respectively). At the same time, there were differences between the Q31L and WT mice in the Int and CUS-2 subgroups. In the first case, Q31L spent less time in the center zone of the OF test compared to the WT (M-U, *p* < 0.05), whereas it was the opposite in the CUS-2 subgroup (M-U, *p* < 0.001); however, changes in the WT mice may have been due to decreased locomotor activity (Figure 2).

Analysis by the Kruskal–Wallis test of common time in the center zone revealed differences between groups within the Q31L genotype (χ^2^ = 12.87; df = 2; *p* < 0.01); mice after CUS-2 spent more time in the center zone than the Int (M-U, *p* < 0.001) and CUS-4 subgroups based on the tendency level (*p* = 0.051).

**Figure 3:**
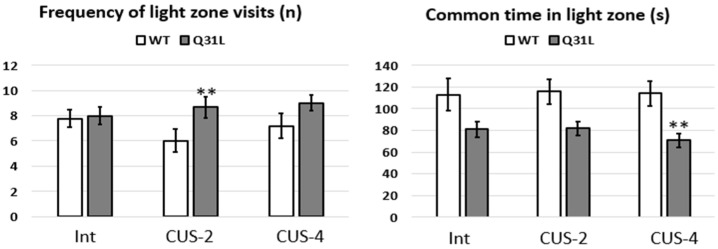
Anxiety related parameters of the DLC test. Data presented as mean ± SE. Int—intact animals, CUS-2—animals after two weeks of CUS, CUS-4—animals after four weeks of CUS; ** *p* < 0.01. Sample size: 8–10 mice in each group.

In the DLC test (Figure 3), the ANOVA analysis of the frequency of light zone visits revealed a significant effect of genotype factor (F [1,49] = 6.35, *p* < 0.01), where there was an increase in this parameter only in the Q31L mice compared with the WT in the CUS-2 subgroups. ANOVA analysis of time in the light zone also revealed the significant effect of genotype factor (F [1,49] = 12.83, *p* < 0.01), but there was a significant decrease in time in Q31L compared with the WT mice in the CUS-4 subgroups. CUS did not affect the mice within genotypes. Thus, the DLC test confirmed the absence of anxiety in both strains of mice, but in Q31L, stress changed the anxiety-related parameters.

**Figure 4:**
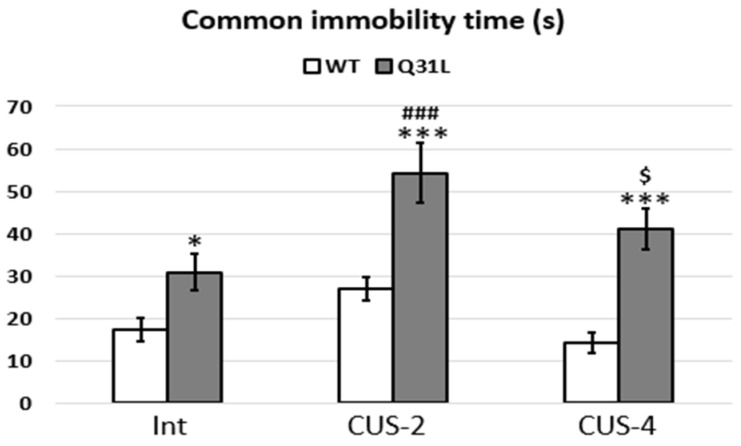
Freezing in the OF test. Data presented as mean ± SE. Int—intact animals, CUS-2—animals after two weeks of CUS, CUS-4—animals after four weeks of CUS; *^,$^ *p* < 0.05, ***^,###^ *p* < 0.001; *—significant difference between genotypes in subgroups, $—significant difference between CUS-2 and -4 inside one genotype. Sample size: 8–10 mice in each group.

Immobility in the OF test is based on behaviors positively correlated with fear or emotionality (Figure 4) [30]. Two-factor ANOVA analysis of the total duration of freezing showed a significant effect of genotype (F [1,51] = 42.35, *p* < 0.001) and stress (F [2,51] = 8.4, *p* < 0.001). The Q31L mice had a higher freezing duration than the WT mice in all subgroups (LSD, *p* < 0.05 for Int; LSD, *p* < 0.001 for both CUS subgroups). An increase in freezing time was shown in the Q31L mice after CUS-2 (LSD, *p* < 0.001), and there was a difference between the CUS-2 and CUS-4 subgroups (LSD, *p* < 0.05), but the last stayed within the intact Q31L range.

It was proposed that the increased time in the center zone after CUS-2 in the Q31L mice may have been due to increased immobility in this zone. However, minute analyses of these parameters during the test and correlation analysis did not reveal any connection in this behavior, and the high frequency and time in the center zone of the OF test may have been due to other behavioral features such as impulsivity.

### 2.3. Two Weeks of CUS Increased Social Motivation in Q31L While It Decreased in WT

**Figure 5:**
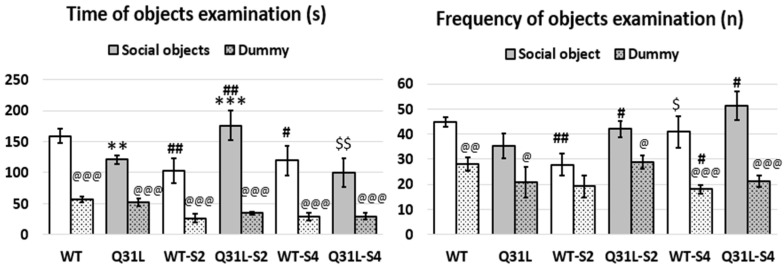
Social preference and social motivation. Data presented as mean ± SE. S2—animals after two weeks of CUS, S4—animals after four weeks of CUS; ^@,#,$^ *p* < 0.05, **^,@@,##,$$^ *p* < 0.01, ***^,@@@^ *p* < 0.001; #—significant difference between CUS subgroups and Int inside one genotype, $—significant difference between CUS-2 and -4 inside one genotype, @—significant difference between social object and dummy. Sample size: 8–10 mice in each group.

In the social test (ST), we observed social preference and social motivation (Figure 5). Repeated measure ANOVA analysis revealed the significant effect of stress (F [2,50] = 3.19, *p* < 0.05), the object of interest (F [1,50] = 197.15, *p* < 0.001), gene–stress interaction (F [2,50] = 4.37, *p* < 0.05), and gene stress–object of interest interaction on the objects’ examination time. No deviation in the social preference according this parameter (LSD, *p* < 0.001 in all groups between social object and dummy) was observed. Repeated measure ANOVA analysis of the frequency of object examination revealed a significant effect of the interest object (F [1,50] = 66.43, *p* < 0.001), gene–stress interaction (F [2,50] = 5.73, *p* < 0.01), and stress–object interaction (F [2,50] = 4.59, *p* < 0.05). Accordingly, this parameter was found in the absence of social preference only in the WT mice after CUS-2.

**Figure 6:**
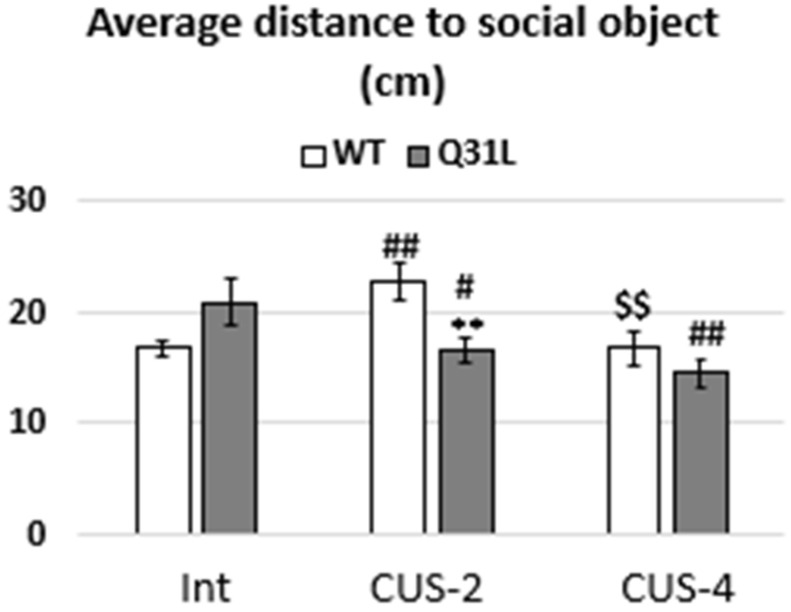
Average distance from mice to the intruder. Data presented as mean ± SE. Int—intact animals, CUS-2—animals after two weeks of CUS, CUS-4—animals after four weeks of CUS; ^#^ *p* < 0.05, **^,##,$$^ *p* < 0.01; #—significant difference between CUS subgroups and Int inside one genotype. Sample size: 8–10 mice in each group.

Post hoc analysis revealed differences in mice social motivation (Figure 6). It was found that the intact Q31L mice spent less time near the intruder than the WT (LSD, *p* < 0.01), but after 2 weeks of CUS, the time of intruder examination increased (LSD, *p* < 0.01), whereas in the WT, it decreased as well as the frequency of examination (LSD, *p* < 0.01) and became lower than Q31L in the same subgroup (LSD, *p* < 0.001 and *p* < 0.05 for time and frequency, respectively). There was no difference between the Q31l and WT mice after CUS-4 in the time and frequency of object examination. After 4 weeks of CUS, the WT mice has still had low social motivation according to the time examination (LSD, *p* < 0.05), whereas the Q31L mice returned to the time of social interaction as that of the intact level (LSD, *p* < 0.01 in comparison with Q31L-S2). At the same time, contrasting changes occurred with the frequency of object examination in the mice after CUS-4: an increase in the WT to the intact mice level (LSD, *p* < 0.05 in comparison with WT-S2) and in Q31L (LSD, *p* < 0.05 in comparison with intact Q31L mice), which may reflect the increased impulsivity in Q31L mice after long-term stress.

Additionally, was assessed the average distance from the mice to the social object (Figure 6). Two-way ANOVA analysis showed a significant effect of stress (F [2,50] = 4.52, *p* < 0.05) and gene–stress interaction (F [2,50] = 6.77, *p* < 0.01). Post hoc analysis revealed that the Q31L mice after CUS-2 were closer to the intruder than the WT in the same subgroup (LSD, *p* < 0.01), in which stress increased the distance (LSD, *p* < 0.01), and the intact Q31L mice (LSD, *p* < 0.05). There was no difference between the Q31L and WT mice in the CUS-4 subgroup, the WT mice returned to the intact level (LSD, *p* < 0.01 in comparison with WT-S2), and the Q31L mice still moved more closely to the intruder than the intact mutant mice (LSD, *p* < 0.01). Together, this shows the aberrant changes in social motivation in the Q31L mice, especially after CUS-2.

### 2.4. CUS Induced Depressive-Like Behavior in WT Mice but Did Not Affect Depressive-Like Behavior in Q31L Mice

**Figure 7:**
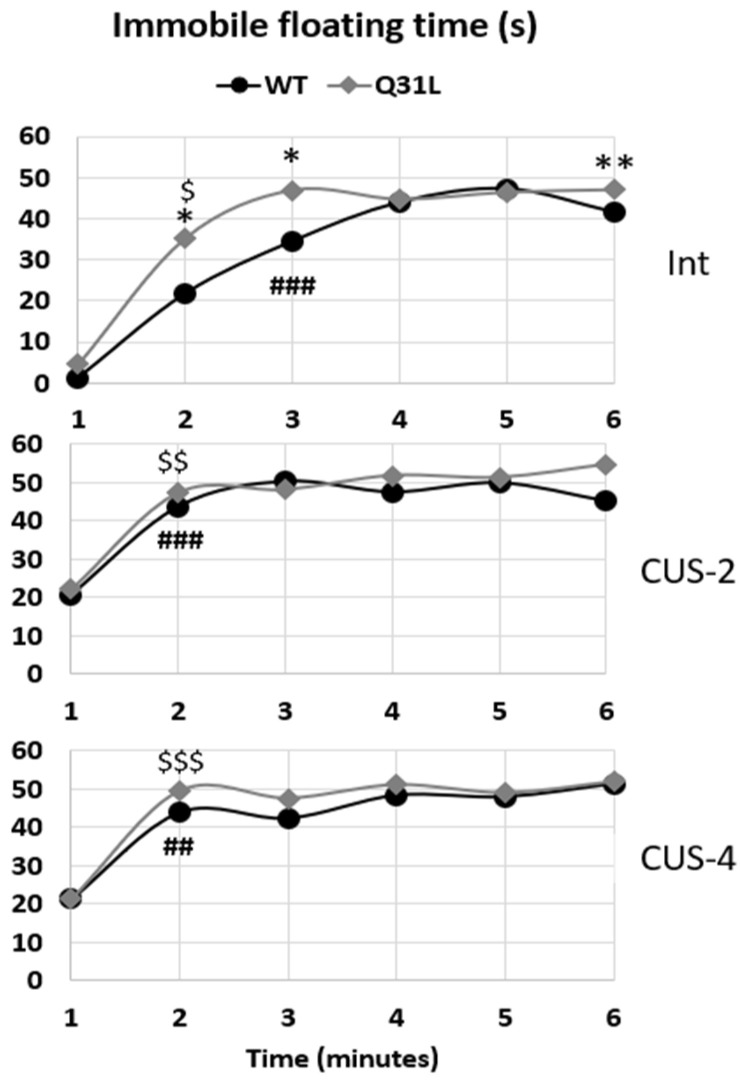
Minute immobility changes in the FST. Data presented as median. Int—intact animals, CUS-2—animals after two weeks of CUS, CUS-4—animals after four weeks of CUS; *^,$^ *p* < 0.05, **^,##^ *p* < 0.01, ^###,$$$^ *p* < 0.001; *—significant difference between genotypes in subgroups, $—first time of high immobility level compared with the first minute in the Q31L group. Sample size: 8–10 mice in each group.

Immobility in the forced swim test (FST) is a classical marker of depressive-like behavior in rodents. Using the Freedman repeated measure test, it was shown that there were minute changes in mice immobility/floating in all groups (*p* < 0.001). The W-test revealed that the intact Q31L mice reached a high immobility level at the second minute of the test (W, *p* < 0.05), whereas the WT mice became immobile at the third minute of the test. After 2 and 4 weeks of CUS, high immobility was reached at the second minute in both strains of mice (Figure 7).

The M-U test revealed a higher immobility time in the intact Q31L mice compared with the intact WT mice at the second, third (M-U *p* < 0.05), and sixth minute of the test (M-U *p* < 0.01), but in the CUS-2 and CUS-4, there were no differences between strains.

**Figure 8:**
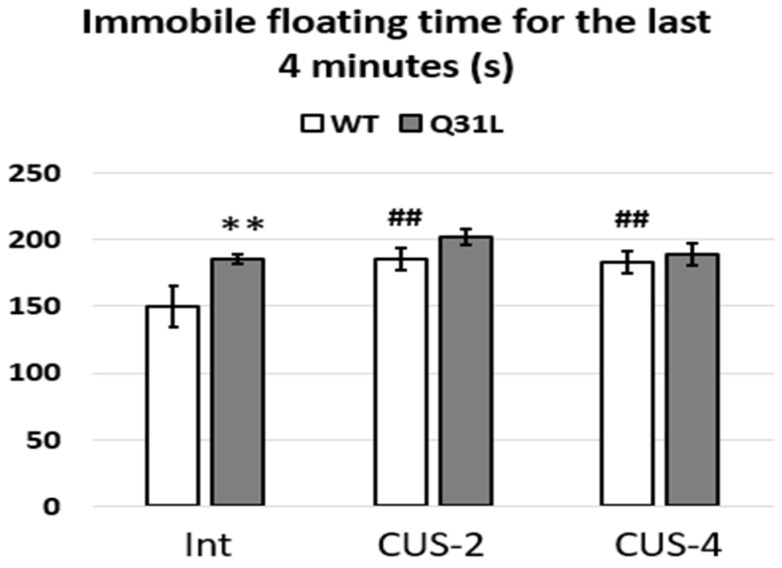
Common immobile floating during last 4 min of the FST. Data presented as mean ± SE. Int—intact animals, CUS-2—animals after two weeks of CUS, CUS-4—animals after four weeks of CUS; **^,##^ *p* < 0.01. Sample size: 8–10 mice in each group.

ANOVA analysis of immobile floating for the last 4 min of the test (Figure 8) revealed a significant effect of genotype (F [1,49] = 8.17, *p* < 0.01) and stress (F [2,49] = 5.46, *p* < 0.01). It was shown that the Q31L mice had depressive-like behavior in the FST (LSD, *p* < 0.01) that did not change after 2 and 4 weeks of CUS, whereas the WT after stress showed increased immobility in the test (LSD, *p* < 0.01, for both CUSs).

### 2.5. CUS Did Not Increase the Plasma Corticosterone Level and Not Induce Body Weight Loss in Q31L Mice

**Figure 9:**
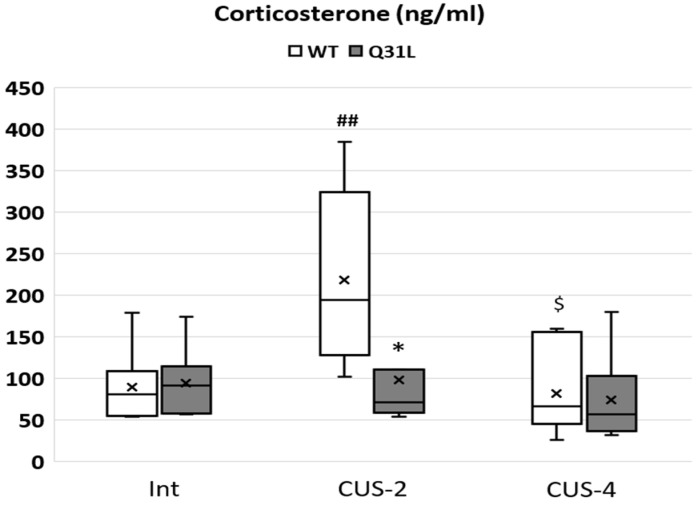
Plasma corticosterone level. Data presented as median, Q1 and Q3, minimum and maximum as whiskeys; X—mean. Int—intact animals, CUS-2—animals after two weeks of CUS, CUS-4—animals after four weeks of CUS; *^,$^ *p* < 0.05, ^##^ *p* < 0.01; *—significant difference between genotypes in subgroups, $—significant difference between CUS-2 and -4 inside one genotype. Sample size: 7–8 mice in each group.

Increased corticosterone level is the main sign of stress in animals including humans and rodents. The Kruskal–Wallis test revealed significant changes between groups (Figure 9) inside the WT genotype (χ^2^ = 11.4; df = 2; *p* < 0.05). There was no difference in the plasma corticosterone level between the WT and Q31L in the Int subgroup, but after 2 weeks of CUS, in the WT mice, there was a significantly higher level of this hormone in comparison with the intact WT (M-U, *p* < 0.01) and with the Q31L mice in the CUS-2 subgroup (M-U, *p* < 0.05). After 4 weeks of CUS, the corticosterone level in the WT mice returned to the normal level (M-U, *p* < 0.05 in comparison with WT-S2).

**Figure 10:**
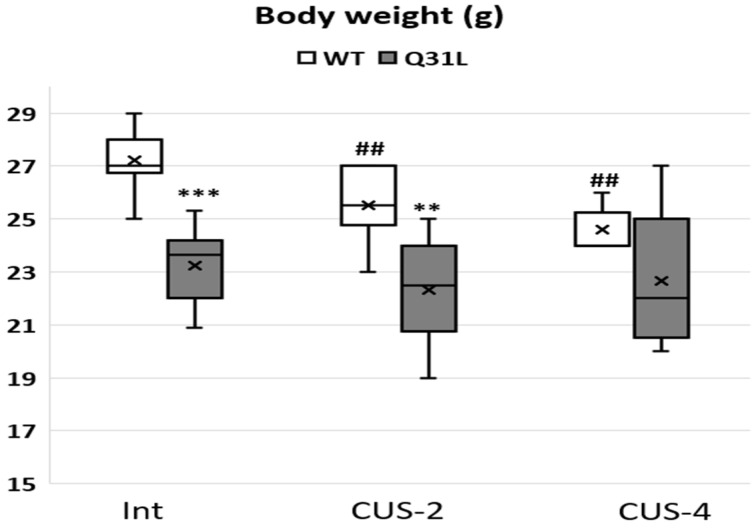
Body weight changes in mice after CUSs. Data presented as median, Q1 and Q3, minimum and maximum as whiskeys; x—mean. Int—intact animals, CUS-2—animals after two weeks of CUS, CUS-4—animals after four weeks of CUS; **^,##^ *p* < 0.01, *** *p* < 0.001. Sample size: 8–10 mice in each group.

Stress is also accompanied by body weight changes; in rodents, it is usually weight loss [31]. The Kruskal–Wallis test revealed significant changes between groups (Figure 10) only inside the WT genotype (χ^2^ = 14.9; df = 2; *p* < 0.001). After 2 and 4 weeks of CUS, there was a significant decrease in body mass in the WT mice (M-U, *p* < 0.01). The Q31L mice had a significantly lower body mass in the Int (M-U, *p* < 0.001) and CUS-2 (M-U, *p* < 0.01) subgroups in comparison with the WT mice. In contrast with the WT, stress did not induce body weight loss in the Q31L mice.

### 2.6. Q31L Mutation Decreased BMAL1 Immunofluorescence in Key Brain Regions Involved in Affective Disorders and Stress Response

**Figure 11:**
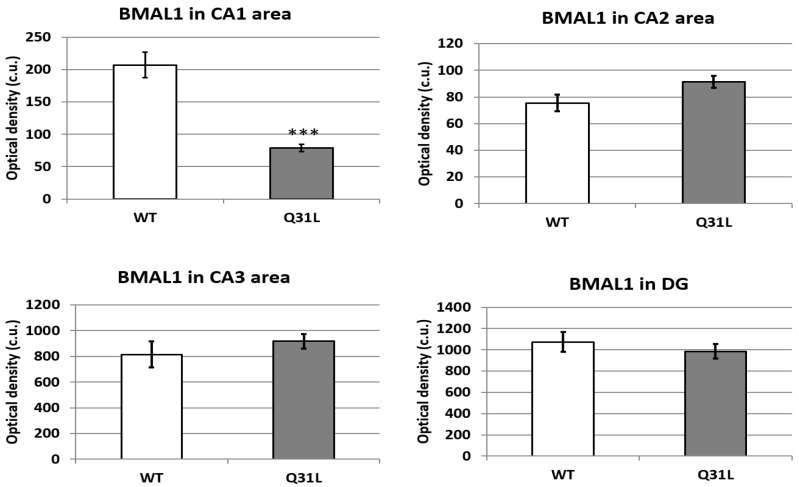
BMAL1 immunofluorescence in the hippocampal area. Data presented as mean ± SE. *** *p* < 0.001. Sample size: 5 mice of each strain.

**Figure 12:**
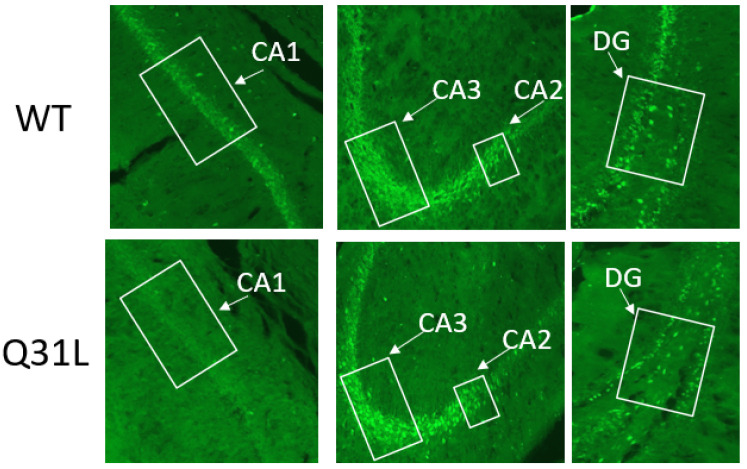
Microphotography of the hippocampal area, zoom 20×. DG—dentate gyrus. White boxes—frame where data were obtained.

BMAL1, aside from its circadian function, is a modulator of stress response on the DNA level [28,29]. Using immunohistochemical (IHC) analysis, we investigated BMAL1 changes in some brain regions involved in affective disorders in Q31L mice. One-way ANOVA analysis of BMAL1 immunofluorescence in the hippocampal area, which may inhibit the HPA axis [20], revealed a significant effect of genotype only in the CA1 area (F [1,39] = 63.98, *p* < 0.001). This was shown to decrease BMAL1 in this area in the Q31L mice (Figure 11 and Figure 12).

**Figure 13:**
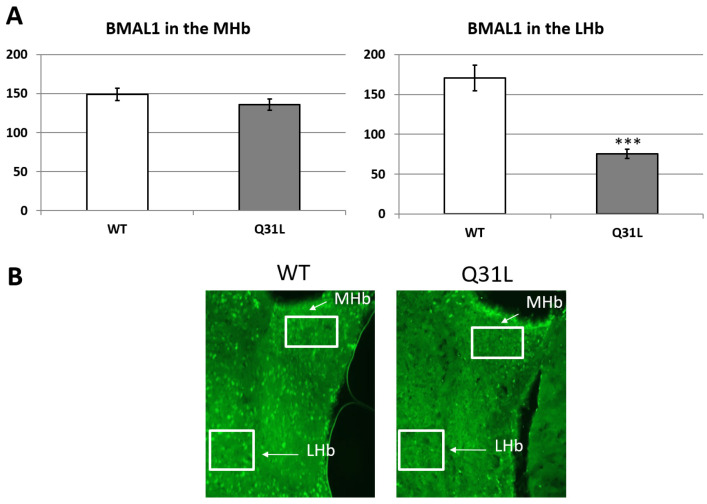
(**A**) BMAL1 immunofluorescence in the habenular region. Data presented as mean ± SE. *** *p* < 0.001. Sample size: 5 mice of each strain. (**B**) Microphotography of the habenular area, zoom 20×. MHb—medial habenula, LHb—lateral habenula. White boxes—frame where data were obtained.

The habenula is important for regulating affective behavior, motivation, and stress response [21]. ANOVA analysis revealed the effect of Q31L genotype (F [1,15] = 34.27, *p* < 0.001) on BMAL1 immunofluorescence in the lateral, but not in the medial habenula (LHb and MHb) and a significant decrease in BMAL1 was seen in this area (Figure 13).

We also assessed the BMAL1 immunofluorescence in the amygdalar complex, which may activate the HPA axis, and in the SCN, which is involved in tonic diurnal regulation of the HPA axis activity [20]. However, there were no differences in BMAL1 between the WT and Q31L mice in these areas.

## 3. Discussion

The interaction of negative genetic and environmental factors has a high influence on the development of affective disorders. Genetic predisposition to dysregulation of the stress response can significantly reduce the adaptive potential of the organism and decrease quality of life [32]. In this article, we identified features of the behavioral and physiological response of mice with the point Q31L mutation in the *Disc1* gene, which are a genetic model of depressive-like behavior [14]. It was shown that Q31L, in contrast to the WT animals, did not experience a stress-associated increase in corticosterone levels in peripheral blood under 2 weeks of CUS condition and weight loss after both CUSs, which reflect a physiological impairment of the stress response. Furthermore, the Q31L mutation changed the behavioral stress response mainly after two weeks of CUS.

In contrast to the WT mice, stress did not change the locomotor activity in Q31L and stayed at a low level, whereas after 2 weeks of CUS, the level of locomotor activity was reduced in the WT mice and reached the Q31L level. This result of the WT in a number of studies can be explained by apathy and reduced motivation, which are signs of depressive-like behavior [33,34]. It was confirmed by decreased sociability and increased immobility time in the FST after 2 weeks of CUS, which reached the intact Q31L level. In contrast to the WT, after 2 weeks of CUS, the Q31L mice showed increased social motivation, but stress did not change the increased immobility in the FST of these mice.

There were some unusual results in the anxiety-associated behavior of Q31L mice after stress, especially after 2 weeks of CUS. Intact Q31L mice showed signs of anxiety-like behavior in the OF test, but after 2 weeks of CUS in OF, an increased frequency of visits, time in center zone, as well as higher rate of light chamber visits in DLC test were shown. This most likely reflects the stress-induced impulsivity in Q31L and can also be confirmed by the higher frequency of close interaction with the social object. After 4 weeks of CUS, only an increased frequency of center zone and social object interaction were observed. Recently, impulsivity has been observed only in female Q31L mice, but there are no data regarding this behavior in male mice, so specific tests will be required in the future study [15]. On the other hand, 2 weeks of CUS increased the immobility time in the OF test, which may reflect fear or an emotionality condition [30]. At the same time, in the WT mice, there were no signs of anxiety, impulsivity, or emotionality after CUS. In some earlier studies, specific tests also did not show signs of anxiety in the WT animals [35,36].

In this work, we assumed that in mice with mutations in the second exon of the Disc1 gene, there possibly exists a disruption of the BMAL1 protein, which inhibits glucocorticoids-mediated activation of gene expression and communicates with the DISC1 interactome through GSK-3 [24,29].

First of all, BMAL1 is a positive regulator of circadian rhythm, which binds to the E-boxes on clock regulated genes and activates its transcription including genes that are involved in HPA axis activity and stress response (*Fkbp5*, *Avp* etc.) [25,26] Furthermore, BMAL1 also performs a number of important functions throughout the body, for example, it protects cells from oxidative stress and early aging, maintains genome stability, exhibits antitumor activity, reduces inflammation, and participates in metabolism and endocrine functions. Moreover, BMAL1 plays a direct role in the development and functioning of the nervous system in both the neuron and glial cells, varying in different brain regions [37,38]. In addition, BMAL1 plays an important role in the survival of brain neurons and through the regulation of microglia-mediated neuroinflammation [39].

Based on such a variety of functions, mutations or disruptions of BMAL1 may be predictors of pathologies including human affective and stress-related disorders. [40]. For example, it was shown that rs7107287 polymorphism in the *BMAL1* gene was associated with cyclothymic temperament, symptoms of depression and stress, and the negative impact of seasonality on well-being throughout the year in humans [41]. Studies on experimental animals with a disruption of BMAL1 have also shown phenotypes similar to human psychopathologies and dysfunction of the HPA axis. *Bmal1* knockout in *Macaca fascicularis* disrupted the production of steroid hormones and increased the level of cortisol in blood. Systemic inflammation was also observed, accompanied by anxiety, depressive-like behavior, and impaired sensory gating [42]. SCN-specific knockout in mice leads to depressive-like phenotype, increased body weight, abnormal circadian rhythm of corticosterone synthesis, and its weak response to stress [43]. Full body *Bmal1* knockout in mice leads to anhedonia, hypocortisolism, and weak response to acute stress due to the suppression of gene transcription involved in cholesterol transport in adrenal cells, which leads to an impaired response of the adrenal glands to ACTH [28]. Thus, the function of BMAL1 as a player of stress response becomes obvious, and this can be proven by the fact that BMAL1 is directly involved in the modulation of the sensitivity of glucocorticoid receptor target genes, physically binding to GRE-promoters and preventing glucocorticoid-mediated transcription [29].

As above-mentioned, BMAL1 is also involved in the synthesis of corticosterone in adrenal cells [27,28]. In our study, behavioral disturbances in the Q31L mice after CUS were not accompanied by changes in the plasma corticosterone levels, so we can conclude that an abnormal behavioral response to stress is due to changes in the functioning of signaling pathways in key brain areas regulating the behavior and stress response of these animals. Mice with L100P point mutation and also disrupted DISC1 N-terminus interactome, had normal corticosterone response to acute stress, so it may reflect that only long duration of aversive events may decrease hormonal stress response in mice with point mutation in the second exon of *Disc1* gene [44]. According to the literature data from *Bmal1*-knokout mice, a deficit in this protein leads to hypocortisolism in physiological conditions [23], whereas in Q31L mice, the basal level of corticosterone in the intact state was within the normal range. Therefore, we propose that disruption of the HPA axis in Q31L mice occurs in brain regions. However, after CUS, BMAL1 may also be disrupted in the adrenal cortex, which can explain the lack of corticosterone response to stress in the Q31L mice. To further test this hypothesis, it would be interesting to study the response of CRH and ACTH to stress in Q31L mice. On the other hand, after stress, the rhythm of corticosterone synthesis may be disrupted [45]. In our study, in the intact Q31L mice, we did not observe BMAL1 changes in the SCN, the master clock of organisms, which may directly regulate the clock activity of the HPA axis [20]. Although some studies have reported the effects of stress on the SCN clock in adult animals, others have found that the clock is rather insensitive to glucocorticoids due to the absence of its receptors. This mechanism may provide stability of the clocks from unexpected fluctuations in the glucocorticoid levels [46]. However, BMAL1 changes in the SCN in Q31L mice after stress still need to be studied.

Another reason for the absent hormonal stress response of Q31L mice may be due to disrupted DISC1–PDE4B interaction, which leads to a decrease in PDE4B activity [14,47]. According to earlier studies, CUS promotes an increase in the amount of PDE4D and PDE4B; moreover, the use of the PDE4 family inhibitor rolipram reduces the stress-induced increase in corticosterone levels in the blood and restores the behavior of stressed animals. Thus, attenuated PDE4B activity in the Q31L mice may explain the less pronounced hormonal stress response in Q31L mice [48,49]. In WT mice, CUS increased the blood corticosterone level, which is in agreement with early data, suggesting that a shorter CUS has a more pronounced effect on mice [31]

The hippocampus is a brain region that indirectly inhibits the activity of the HPA axis. It has been shown that stimulation of its CA1 zone reduces activity of the hypothalamic periventricular nucleus, which accumulates signals from brain structures and activates the HPA axis, thus reducing glucocorticoid secretion [20]. In the hippocampus, BMAL1 has an influence on the adult brain neurogenesis processes, regulates dopamine signaling, and establishes daily oscillations in cAMP levels and MAPK signaling activity, thereby participating in the formation of long-term signal potentiation and hippocampus-dependent memory [50,51,52]. BMAL1 is also localized to CA1 pyramidal neurons of the hippocampal region, where it regulates synaptic plasticity in a circadian manner through interaction with calmodulin-dependent kinase 2α [53].

In this study, we observed that the intact Q31L mice had a lower BMAL1 immunofluorescence in the CA1 zone than the WT animals. In our resent study, Q31L mice in the passive avoidance task showed an increase in the fear extinction time, which may be a sign of the impaired formation of a new memory trace and aberrant reaction on acute stress [16]. The CA1 zone is also a key region involved in the pathogenesis of one of the most well-known stress-related diseases—post traumatic stress disorder (PTSD)—and the increase in the fear extinction time corresponds to the symptom of re-experiencing in this disorder, thereby indicating the predisposition of mice with the Q31L mutation in the *Disc1* gene to PTSD-like behavior including due to BMAL1 dysregulation [54,55]. The role of BMAL1 in this behavior is also supported by the fact that polymorphism of the *RORα* gene, whose protein product activates *BMAL1* transcription, is a risk for the development of PTSD in combat veterans [56].

LHb is a key regulatory area of motivation, a critical region for recognizing signals of punishment and reward, and is involved in the pathogenesis of affective disorders including depression and bipolar disorder [15]. Recent studies have shown the role of this region in the stress response, since the LHb is activated when exposed to almost all types of stressors [21]. Acute stress transforms LHb signals in such a way that reward leads to activation of this structure, similar to punishment or missed reward, which can disrupt the perception of reward signals and contribute to the development of depression under stress [57]. Moreover, LHb is able to receive feedback signals from the hypothalamus, and potentiation of these synaptic connections can cause depression under stress conditions [58]. A BMAL1 decrease in Q31L mice LHb can lead to disruption of the motivation, which is expressed as a decrease in motor and exploratory activity in intact mice as well as the time of social interaction, and to a pronounced depressive-like phenotype in Q31l animals. Apparently, disruption of BMAL1-dependent signaling pathways in this area led to an increase in the time of social interaction after 2 weeks of CUS in Q31L mice as well as the appearance of impulsivity signs [59,60]. Interestingly, despite the reduced expression of BMAL1 protein in the LHb of Q31L mice, they were previously shown to have an increase in neuronal density in the lateral and medial regions of the habenula, suggesting that the decrease in BMAL1 in this region may not be associated with neurons, but with glial cells, the violation of which leads to depressive-like behavior, especially if it disrupts the myelinization process [15,61,62]. A decrease in LHb BMAL1 may also contribute to the disruption of processes associated with the memory of aversive events and avoidance behavior, since the LHb plays a key role in them [63,64].

Thus according our data and recent studies about the role of BMAL1 in the stress reaction, we propose that BMAL1 dysregulation, caused by the disruption of the DISC1 N-terminus in CA1 and LHb mice brain areas, may disrupt the behavioral stress response in mice, which may be one of the key molecular mechanisms underlining affective and stress-related disorders.

***Limitations.*** The study of the BMAL1 protein content in various brain structures of Q31L mice was conducted for the first time, and the first quantitative results were obtained using the IHC method. The impact of stress was assessed using labor-intensive behavioral tests. All work required a fairly large number of mice, both wild type and Q31L mutants. The number of mice that could simultaneously participate in the experiment was limited and depended on the conditions of their breeding. Therefore, our conclusions regarding the participation of the BAMAL1 protein in the stress response in mutant mice were based mainly on the literary data. In order to prove the hypothesis, we have put forward another large experiment that will be prepared. We are planning to conduct a study of BMAL1 in brain structures after stress using semi-quantitative methods to determine the levels of the BMAL1 messenger and transcriptional activity of the stress-related genes and study the activity of GSK-3 in BMAL1 phosphorylation with a focus on the stability of BMAL1 by modulation of its ubiquitination.

## 4. Materials and Methods

### 4.1. Animals

Male mice of two genetic strains were used in this work: C57BL/6 (WT) and homozygous Q31L mice. Animals were obtained from the unique scientific installation “Biological collection—genetic biomodels of neuropsychiatric diseases” (No. 493387) of the Research Institute of Neuroscience and Medicine (Novosibirsk, Russia) [26]. For the behavioral study and enzyme-linked immunosorbent assay (ELISA), we used 60 males, 30 per strain, and 10 for the experimental subgroup (see Table 1). For the IHC study, we used 10 animals, with 5 per strain. All mice were 2–4 months old and had weights between 20 and 30 g. The criteria for exclusion from the experiment were visible external damage, disorders of the musculoskeletal system, aggressive behavior (exclusion of all mice in cells from the experiment), severe malnutrition, or obesity. Animals were kept in accordance with standard laboratory care requirements with a light cycle of 12:12 h (lights turn on at 5:00 and turn off at 17:00) and an ambient temperature of 22–25 °C in plastic cages measuring 34 × 29 × 15 cm, no more than five mice in one, with constant access to food and water. Work with animals was carried out in accordance with the bioethical standards of the European Union Directive (ECC Directive 86/609/EEC). The experiments (Figure 1) were approved by the local ethics committee of the Research Institute of Neuroscience and Medicine (protocol No. 5-v dated 04/06/2023).

### 4.2. Chronic Unpredictable Stress Protocol

**Figure 14:**
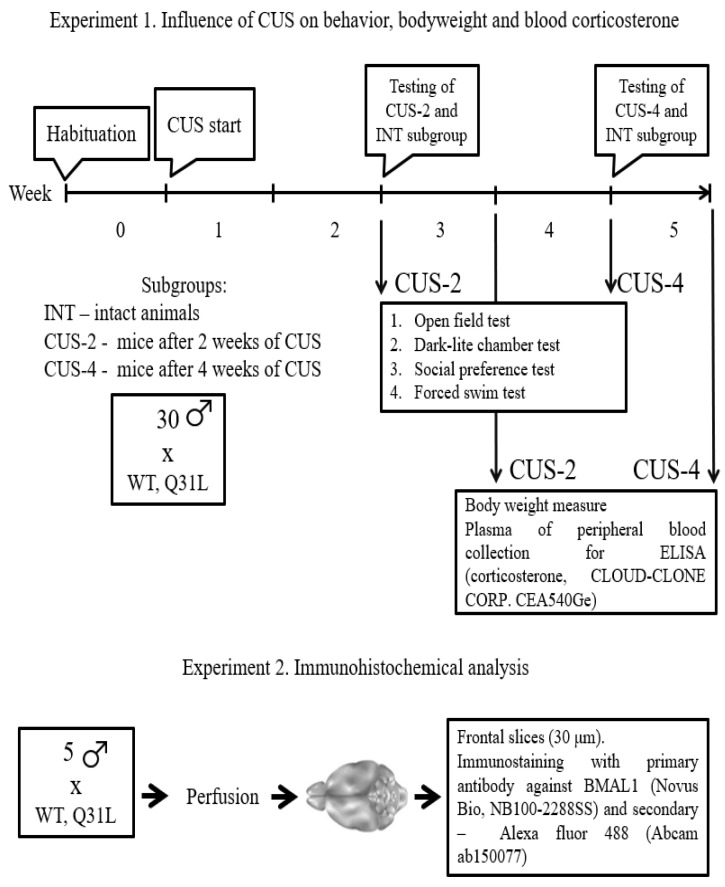
Experimental design. INT—intact animals, CUS-2—animals after two weeks of CUS, CUS-4—animals after four weeks of CUS; in squares—quantity of male mice per each genotype, in rectangles—description of used methods.

**Figure A2:**
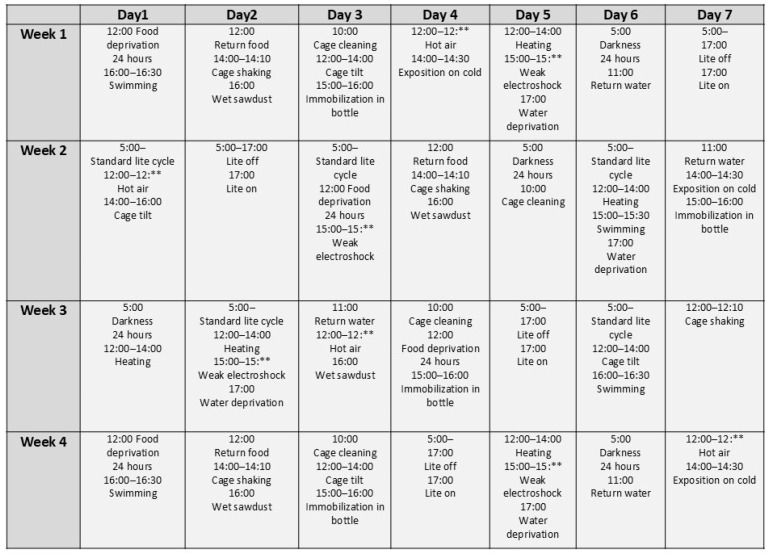
CUS timetable used in the present study. ** is undistinguished time of stressor ending.

To study the effect of stress on the Q31L mice, a chronic unpredictable stress (CUS) protocol was used, which includes several different stress factors presented at different times to solve the problem of adaptation [65,66]. In this work, the CUS protocol was based on previous studies (see, Figure A2) and included the following stress factors: confinement in a bottle for 1 h, rocking the cage for 10 min, water deprivation for 18 h, bathing in a small amount of water for 30 min, wet bedding for 18 h, heating up the cage at 45° for two hours, electric shock (0.1 mA) three times, heating the holding room to 40 °C, hot air three times, exposing cells to cold (+2 °C) for half an hour, darkness for 24 h, and inverted lighting [35,67]. Since there were mixed data on the effect of the duration of the CUS procedure on the behavioral and physiological parameters of the WT mice, we decided to use 2 and 4 week protocols (14 and 28 days) [31,68]. Before the start of stress, animals of each genotype were divided into subgroups (see Table 1): intact control (Int), mice subjected to two-week (S2) and four-week (S4) CUS, 10 animals per subgroup. Mice of the Int subgroup were kept in a separate room and only standard procedures for bedding, cleaning, and feeding were applied. Before the CUS procedure started, mice in subgroups S2 and S4 were transferred to a separate room where were kept under standard conditions for a week for habituation before CUS. On the next day after the end of CUS, the animals were tested in standard behavioral tests. The complete experimental design is presented in Figure 14.

### 4.3. Behavioral Study

All behavioral studies were carried out according to standard protocols in order of least to most stressful effects. The work used automatic tracking and data collection using Ethovision XT10 software and equipment (Noldus Information Technology, Wageningen, The Netherlands). Before the study, the animals were moved to the testing room for 30 min. Behavioral tests were carried out over a period of 3–3.5 h (12:00–15:00/15:30) depending on the duration of one test session, which corresponded to ZT 7–10 (Zeitgeber time, measured relative to the light cycle, where ZT 0 is the time lights on, in our case 5:00). After each mouse was tested, the laboratory equipment was cleaned with 70% ethanol to remove odors.

A standard open field (OF) test was used to assess mice motor and exploratory activity, emotionality, and anxiety animals. A square arena measuring 50 × 50 × 50 cm with transparent plastic walls and an opaque white bottom was used. Recordings were carried out for 10 min, the distance traveled, time in the center of the arena, number of vertical stands, freezing time and frequency, and the number of defecations [69].

The dark–light chamber (DLC) test to assess anxious behavior in rodents was a 22 × 78 × 40 cm plastic setup, divided into two compartments. One third of the total area was occupied by a dark compartment covered with a lid made of lightproof material. The remaining two thirds were occupied by an open compartment illuminated by two directional lamps, with white walls and floor, separated from the dark chamber by a door measuring 7 × 7 cm. Testing was carried out for 5 min. The following parameters were automatically monitored: duration of stay in the white chamber, latent time to exit the black chamber, and average distance from the entrance to the black chamber [70].

The study of the social behavior characteristics of the mice was carried out using equipment for an open field test, and two metal cylinders with a diameter of 8 cm and a height of 15 cm with slots. The test protocol included a 5-min habituation and a 10-min test. After habituation, the mouse was placed in a separate cage, and in the testing chamber, a social object (WT male mouse) was placed under one cylinder, and a non-social object (dummy) was placed under the other, after which the mouse was again released into the arena for 10 min. The most informative parameters were the total time of sniffing objects, average distance from the nose to a social or nonsocial object as well as time spent in the area near each object [71].

The test for assessing depressive-like behavior was a cylinder 50 cm high and 20 cm in diameter with water at room temperature poured to the 37 cm mark. The mouse was placed in water for 6 min. Water in the cylinder was changed after every five mice. The most informative indicator for this test is the floating duration over the last 4 min of the test [72].

### 4.4. Corticosterone Immunoassay

The day after behavioral testing, the animals were weighed, sacrificed by decapitation, and peripheral blood was collected in heparinized tubes. The collection of biomaterials was carried out at the same time as testing (ZT 7–10). The blood was allowed to settle for 30 min, then spun in a centrifuge for 15 min at 3000 rpm, after which the blood plasma was collected and frozen at −70 °C for subsequent enzyme-linked immunosorbent assay (ELISA).

ELISA was performed using the ELISA kit for corticosterone in the blood (CEA540Ge, CLOUD-CLONE CORP, Wuhan, China) according to the standard method from the manufacturer using the iMark^TM^ device microplate absorbance reader (BIO-RAD, Hercules, CA, USA). The criteria for excluding samples from the analysis were the presence of hemolysis, turbidity, or the appearance of clots in the tube. After selection, there were 47 samples in common.

To obtain information about the content of corticosterone in the blood, the results of light absorption signal were converted into concentration values (ng/mL). Then, based on the standard curve data, the most accurate approximation curve was selected (assessed by the approximation reliability value R^2^), a regression equation was obtained, and the corticosterone concentration was calculated using Equation (1):

y = −2.3575 × 3 + 5.3842 × 2 − 5.4077x + 3.2772 (R^2^ = 0.9991) (1)

where x is the absorption value of the light signal of the sample; y is the concentration of corticosterone; R^2^ reflects the reliability of the approximation.

### 4.5. Immunohistochemical Analysis

Animals of each genotype (5 mice per group) from the Int subgroup were used for immunohistochemical analysis (IHC). Mice were anesthetized using CO_2_ after transcardial perfusion was performed: first, the blood was washed out using phosphate-buffered saline (assessed by liver lightening), and then the tissues were fixed with a 4% formaldehyde solution. The procedure took place during ZT 7–8. After this, the brain was carefully removed and placed in a solution of 30% sucrose with the addition of 4% formalin and fixed for a month at a temperature of 4 °C. After this time, the brain was frozen in a special Tissue-Tek O.C.T. medium compound (Sakura Finetek, Torrance, CA, USA) and stored at −72 °C.

Frontal sections with a thickness of 30 μm were obtained using a MicroCut-SADV cryostat (Citotest Labware Manufacturing Co., Haimen, China). The boundaries of the anatomical structures of the mouse brain were determined using the atlas: SCN (Bregma: from −0.23 mm to −0.83 mm), hippocampus, habenula, and amygdala complex (Bregma: from −1.67 mm to −2.45 mm) [73]. IHC was performed using standard frozen section techniques using primary antibodies against BMAL1 (1:800, NB100-2288SS, NovusBio, Englewood, CO, USA) and fluorescently tagged Alexa Fluor 488 secondary antibodies (1:600, ab150077, Abcam, Cambridge, MA, USA).

To obtain micrographs of the brain structures, an AxioPlan 2 imaging fluorescent microscope (ZEISS, Oberkochen, Germany) was used. Image analysis was performed using the Image-Pro Plus 6.0 program (Media Cybernetics, Washington, MD, USA). Data were obtained as the optical density of immunofluorescence in identical sections of micrographs for each structure, subtracting the staining signals of non-immunoreactive areas.

### 4.6. Statistical Analysis

All data obtained were subjected to statistical analysis using Jamovi 2.4.8. software for Windows (retrieved from https://www.jamovi.org, access date: 24 August 2023). The normality of distribution was assessed using the Shapiro–Wilk test. For samples with normal data distribution, one-way or two-way ANOVA for independent samples was used, where the influence of genotype and stress factors was studied. Repeated measures ANOVA was used for dependent samples. Post hoc analysis was performed using Fisher’s test (LSD). For independent samples whose distribution did not correspond to normal, the Kruskal–Wallis test was performed as a non-parametric analog of one-way ANOVA, followed by the Mann–Whitney test (M-U) for pairwise comparisons; in the case of dependent samples, the Friedman test followed by the Wilcoxon test (W) were used.

## 5. Conclusions

Point mutation Q31L located in the second exon of the *Disc1* gene leads to disrupted physiological and behavioral stress response in male mice. We proposed a new possible mechanism of this gene–environment interaction through the DISC1-GSK3-BMAL1 pathway. Disturbances of the BMAL1 protein in brain regions regulating the HPA axis and involved in affective disorders in male Q31L mice confirm the role of the DISC1 N-terminal domain in the stabilization of BMAL1. Moreover, given the known neuroprotective properties of BMAL1 and its ability to inhibit glucocorticoid-mediated gene activation, its reduction in brain regions in the Q31L mice is likely to be associated with an impairment in their physiological stress response and changes in behavioral response to chronic stress. In summary, this study confirms the link between DISC1, BMAL1, and the stress response, and although the precise mechanism remains to be explored, these findings open the door to exploring BMAL1 as a therapeutic target for the treatment of stress-related affective disorders.

## Figures and Tables

**Table 1:** Mice in the experimental groups.

Mice (60 Animals)	WT (30 Animals)			Q31L (30 Animals)		
Keeping conditions	Int	CUS-2	CUS-4	Int	CUS-2	CUS-4
Abbreviations	Int—intact animal as control CUS-2—two-week CUS CUS-4—four-week CUS 8–10 mice per group					

## Data Availability

The data presented in this study are available on request from the corresponding author.
